# Insights from Dysregulated mRNA Expression Profile of *β*-Cells in Response to Proinflammatory Cytokines

**DOI:** 10.1155/2022/4542487

**Published:** 2022-01-22

**Authors:** Zhen Wang, Kunlin Huang, Jing Xu, Jia Liu, Ying Zheng

**Affiliations:** ^1^Department of Metabolism and Endocrinology, The Second Xiangya Hospital of Central South University, Changsha, Hunan 410011, China; ^2^Key Laboratory of Diabetes Immunology (Central South University), Ministry of Education, Changsha, Hunan 410011, China; ^3^National Clinical Research Center for Metabolic Diseases, Changsha, Hunan 410011, China; ^4^Center for Medical Research, The Second Xiangya Hospital of Central South University, Changsha, Hunan 410011, China; ^5^Department of Metabolism and Endocrinology, The First People's Hospital of Pingjiang, Pingjiang, Hunan 414500, China

## Abstract

Type 1 diabetes mellitus (T1DM) is a chronic autoimmune disease that is characterized by autoimmunity and its mediated *β*-cell damage. Chronic exposure of *β*-cells to proinflammatory cytokines is known to regulate the expression of many genes, subsequently resulting in the impairment of some signaling pathways involved with insulin production and secretion and/or *β*-cell apoptosis. In our study, RNA sequencing technology was applied to identify differentially expressed mRNAs in MIN6 cells treated with a mix of cytokines, including IL-1*β*, TNF-*α*, and IFN-*γ*. The results showed 809 upregulated and 946 downregulated protein-coding mRNAs in MIN6 cells upon the stimulation of cytokines. Gene Ontology (GO) and Kyoto Encyclopedia of Genes and Genomes (KEGG) biological pathway analyses were performed to predict the functions of dysregulated genes. The networks of circRNA-mRNA were constructed between differentially mRNAs and dysregulated expressed circRNAs in our previous study. In addition, we selected 8 dysregulated mRNAs for further validation by quantitative real-time PCR. The RNA sequencing data showed 809 upregulated and 946 downregulated protein-coding mRNAs. GO analysis showed that the top 10 significant “biological processes,” “cellular components,” and “molecular functions” for upregulated mRNAs include “immune system process,” “inflammatory response,” and “innate immune response” and the top 10 for downregulated mRNAs include “cell cycle,” “mitotic cytokinesis,” and “cytoplasm.” KEGG analysis showed that these differentially expressed genes were involved with “antigen processing and presentation,” “TNF signaling pathway” and “type 1 diabetes,” “cell cycle,” “necroptosis,” and “Rap1 signaling pathway.” We also constructed the networks of differentially expressed circRNAs and mRNAs. We observed that upregulated circRNA 006029 and downregulated circRNA 000286 and 017277 were associated with the vast majority of selected dysregulated mRNAs, while circRNA 013053 was only related to the protein-coding gene, *Slc7a2*. To the summary, these data indicated that differentially expressed mRNAs may play key or partial roles in cytokine-mediated *β*-cell dysfunction and gave us the hint that circRNAs might regulate mRNAs, thereby contributing to the development of T1DM. The current study provided a systematic perspective on the potential functions and possible regulatory mechanisms of mRNAs in proinflammatory cytokine-induced *β*-cell destruction.

## 1. Introduction

Type 1 diabetes mellitus (T1DM) is one chronic autoimmune disease characterized by selective destruction of pancreatic *β*-cells driven by autoimmunity, thereby leading to absolute lack of insulin secretion and hyperglycemia [1]. During the initial stage of the disease, islets of Langerhans are infiltrated by T cells (including CD8+ and CD4+ lymphocytes), B lymphocytes, NK cells, macrophages, and dendritic cells [1]. These immune cells secrete several proinflammatory cytokines when stimulated by target cells or pathogens, such as IL-1*β*, TNF-*α*, and IFN-*γ*, which, however, may have direct deleterious effects on pancreatic *β*-cells. Prolonged exposure of pancreatic *β*-cells to proinflammatory cytokines will not only merely impair *β*-cell insulin production but also induce *β*-cell apoptosis [2]. Therefore, investigating the deeper mechanisms of this process will help us to clarify the pathogenesis of T1DM to develop novel preventive strategy and therapies of the disease.

Chronic exposure of *β*-cells to proinflammatory cytokines is known to regulate the expression of many genes, subsequently resulting in the impairment of several signaling pathways involved in insulin production and secretion and/or *β*-cell apoptosis [1, 3, 4]. Previous studies have demonstrated that cytokines modified the expression of numerous genes which are involved in inflammatory responses (i.e., *Ccl2*, *Cxcl1*, *Cxcl2*, *Icam1*, and *IL15*), IFN-*γ* signaling (i.e., *Irf1*, *Irf7*, *Igtp*, *Stat1*, *Stat2*, *Stat3*, *Stat4*, and *Jak3*), NF-*κ*B regulation (i.e., *Nfkbia*, *Nfkb2*, and *Nfkbiz*), and endoplasmic reticulum (ER) stress and apoptosis (i.e., *Atf3*, *Atf6*, *Atf2*, *Bid*, *Bik1*, *Casp1*, *Casp4*, and *Chop*) [5].

Upon being activated by proinflammatory cytokines, expression of downstream genes is modulated by many pathways, such as NF-*κ*B, STAT3, and ER stress, ultimately leading to *β*-cell damage [6]. Meanwhile, a group of noncoding transcripts, including mircoRNAs (miRNAs), long noncoding RNAs (lncRNAs), and circular RNAs (circRNAs), have also been elucidated to participate in regulating the gene expression in the process of inflammation-induced pancreatic *β*-cell destruction [7–11]. Roggli et al. reported a repertoire of dysregulated miRNAs, including miR-21, miR-34a, miR-146a, and miR-29 families, contributing to the development of T1DM via regulating multiple genes involved with *β*-cell function and apoptosis [12, 13]. Our previous findings also demonstrated that miR-101a and miR-30b contributed to cytokine-mediated *β*-cell dysfunction and apoptosis by targeting antiapoptotic gene Bcl2 and insulin synthesis key gene Neruod1 [14]. lncRNAs are also important modulators in pancreatic *β*-cell function, survival, and apoptosis via regulating gene expression and stabilization [7, 9, 15]. lnc13 was reported as a significant lncRNA to regulate human pancreatic *β*-cell inflammation by allele-specific stabilization of STAT1 mRNA in T1DM [16]; lncRNA MEG3 can regulate *β*-cell identity and function [17]; PLUT has been demonstrated to serve a critical role in regulating transcription of PDX1, a key transcriptional regulator in pancreatic *β*-cell [18]. A series of dysregulated lncRNAs have also been identified in *β*-cell stimulated with a mix of cytokines, which gave a hint that lncRNAs might play an important role in proinflammatory cytokine-induced *β*-cell destruction [5, 19]. circRNAs are another novel subset of noncoding RNAs (ncRNAs) that can regulate gene expression as well as participate in many biological and pathological processes [20, 21]. circHIPK3 exerts its function by sequestering a group of miRNAs, including miR-124-3p and miR-338-3p and by regulating the expression of several pivotal *β*-cell genes, such as Slc2a2, Akt1, and Mtpn; ciRS-7/CDR1as were also proved to be downregulated in the islets of diabetic db/db mice, leading to decreased insulin secretion, reduced *β*-cell proliferation, and survival [22]. Our previous finding has corroborated that a series of circRNAs is dysregulated in pancreatic *β*-cell destruction induced by proinflammatory cytokines. Among them, circRNA 000286 and 017277 have been clarified to regulate insulin biosynthesis and *β*-cell apoptosis [23].

Herein, we investigated differentially expressed mRNAs in cytokine-mediated *β*-cell damage. We found that exposure of *β*-cell to proinflammatory cytokines results in altered expression of a series of mRNAs, some of which are associated with *β*-cell function and apoptosis. We also selected some differentially expressed mRNAs from RNA sequencing data and used quantitative real-time polymerase chain reaction (qRT-PCR) assay to validate them. Furthermore, we analyzed and discussed the possible mechanism(s) involved in regulating these genes. In this study, we identified differentially expressed mRNAs regulated by proinflammatory cytokines in pancreatic *β*-cells and investigated the mechanisms that are involved in the regulation of some mRNAs.

## 2. Materials and Methods

### 2.1. Cell Culture and Chemicals

The mouse insulin secreting cell MIN6 was cultured in Dulbecco's modified Eagle's medium (DMEM, Gibco Laboratories, Grand Island, NY, USA) supplemented with 15% heat-inactivated fetal calf serum (FBS, Thermo Fisher Scientific, Waltham, MA, USA), 50 IU/mL penicillin and 50 mg/mL streptomycin (Thermo Fisher Scientific), and 70 mmol/L *β*-mercaptoethanol (Millipore), at 37°C in a humidified atmosphere of 5% CO_2_. Recombinant mouse interleukin-1*β* (IL-1*β*) was obtained from Sigma. IFN-*γ* and TNF-*α* were purchased from R&D Systems.

### 2.2. Differentially Expressed mRNA Screening and Clustering Analysis

MIN6 cells were seeded into 6-well plates and then treated with or without a cocktail of 2.5 ng/mL IL-1*β*, 2.5 ng/mL TNF-*α*, and 25 ng/mL IFN-*γ* for 24 h. The total RNA was extracted from cells by using Trizol reagents (Thermo Fisher Scientific). RNA integrity was tested by denaturing agarose gel electrophoresis, and RNA quantity and quality were assessed by NanoDrop ND-1000. The RNA was then treated with RiboZero Magnetic Gold Kit and NEB Next® Poly(A) mRNA Magnetic Isolation Module to deplete rRNA and enrich mRNA. The RNA was employed for the construction of sequencing library by using KAPA Stranded RNA-Seq Library Prep Kit. The mRNA screening was carried out using Illumina NovaSeq 6000 (Kangchen Inc., Shanghai, China). Image analysis and base calling were performed using Solexa pipeline v1.8. Sequencing quality was examined by FastQC. The differentially expressed mRNAs were screened based on the count value with R package Ballgown, and *P* value < 0.05 and fold change > 1.5 were considered statistically significant. The normalized expression level of each group was further analyzed with hierarchical clustering (HCL), and data was presented by using TreeView software (v. 1.5). The color green represents low expression, while red represents high expression.

### 2.3. Quantitative Real-Time PCR

Total RNA was extracted from MIN6 cells and was treated with RNase-free DNase. It was reversely transcribed by using a reverse reaction kit, in accordance with the manufacturer's instructions (Promega, Madison, WI, USA). The qPCR was performed with a SYBRGreen supermix (Takara, Dalian, China) on a Roche PCR System (Roche, Basel, Switzerland). *β*-Actin was used as an internal control. The mRNA expression level was calculated by using the 2^-*ΔΔ*Ct^ method. All data were shown as a mean ratio of three different independent experiments.

### 2.4. Gene Ontology (GO) and Kyoto Encyclopedia of Genes and Genomes (KEGG) Pathway Analysis

The differentially expressed mRNAs from sequencing data were applied for GO and KEGG analysis by using the Database for Annotation, Visualization and Integrated Discovery (DAVID) and KEGG database. We uploaded upregulated and downregulated mRNAs into the databases, and the enrichment score and Fisher's exact test were obtained. The -log10 (*P* value) indicated the enrichment score, which represents the significance of GO term and KEGG pathway enrichment.

### 2.5. Protein-Protein Interaction (PPI) Networks Construction

We selected top 20 up- and downregulated mRNAs and uploaded them into String (https://www.string-db.org/) for PPI analysis. The PPI networks were constructed based on the online tool.

### 2.6. circRNA-mRNA Coexpression Networks

To further examine the potential relationship between differentially expressed circRNAs and mRNAs in proinflammatory cytokine-induced pancreatic *β*-cell damage, the differentially expressed circRNA-mRNA coexpression networks were constructed. We selected differentially expressed circRNAs in MIN6 cells with the stimulation of cytokines according to a microarray analysis in our previous study. circRNA-mRNA pairs with an absolute value of PCC > 0.95 and *P* < 0.01 were defined as coexpressed circRNA-mRNA pairs. The coexpressed circRNA-mRNA networks were visualized using Cytoscape.

### 2.7. Statistical Analysis

The differentially expressed mRNAs were screened based on the count value with R package Ballgown, and *P* value < 0.05 and fold change > 1.5 were considered statistically significant. The data was analyzed by an *F*-test. Some statistical analyses were conducted by using Graphpad Prism 5.0. The data were shown as the mean ± SD from three independent experiments and were analyzed by Student's *t* or one-way ANOVA test. *P* < 0.05 was considered statistically significant.

## 3. Result

### 3.1. Differentially Expressed mRNAs Were Identified in Pancreatic *β*-Cells Stimulated with Proinflammatory Cytokines

**Figure 1:**
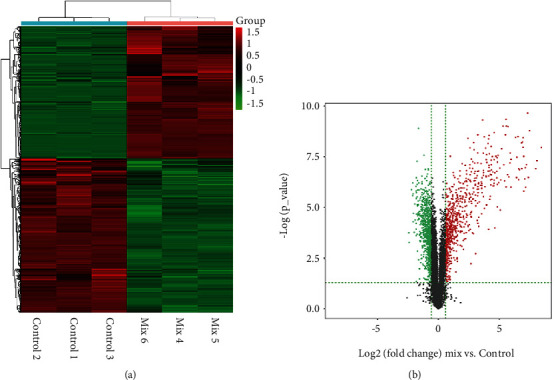
Changed expression pattern of mRNAs in cytokine-mediated pancreatic *β*-cell destruction. (a) The heat map of differentially expressed mRNAs in pancreatic *β*-cells treated with a mix of cytokines, including IL-1*β*, TNF-*α*, and IFN-*γ*, compared with control cells from RNA sequencing data. (b) Volcano analysis showing differentially expressed mRNAs in cytokine-treated pancreatic *β*-cells and control cells.

To identify differentially expressed mRNAs in cytokine-induced pancreatic *β*-cell dysfunction, MIN6 cells were incubated for 24 h with a mix of TNF-*α*, IL-1*β*, and IFN-*γ*. The expression levels of protein-coding genes were determined by RNA sequencing. From the sequencing data, 809 upregulated and 946 downregulated protein-coding mRNAs were obtained. By using R package “heat map,” hierarchical clustering analysis of dysregulated mRNAs was conducted. We also performed the volcano analysis by using Matplotlib. Hierarchical clustering analysis and volcano analysis of up- and downregulated mRNAs are displayed in Figures 1(a) and 1(b). Cytokine-modified expression of numerous protein-coding genes involved in inflammatory responses (i.e., *Ccl2*, *Ccl5*, *Ccl8*, *Ccl20*, *Cxcl10*, *Cxcl2*, and *Icam1*), IFN-*γ* signaling (i.e., *Irf1*, *Irf2*, *Irf5*, *Irf7*, *Igtp*, *Stat1*, *Stat2*, *Stat3*, and *Jak2*), NF-*κ*B regulation (i.e., *Nfkb1*, *Nfkbia*, *Nfkbib*, *Nfkb2*, and *Nfkbiz*), and endoplasmic reticulum (ER) stress and apoptosis (i.e., *Atf3*, *Bid*, *Casp1*, *Casp4*, and *Ddit3*). The top 20 of upregulated and downregulated mRNAs are listed in Table 1.

### 3.2. GO Term and KEGG Pathway Analyses of Differentially Expressed mRNAs in Pancreatic *β*-Cells Stimulated with Proinflammatory Cytokines

**Figure 2:**
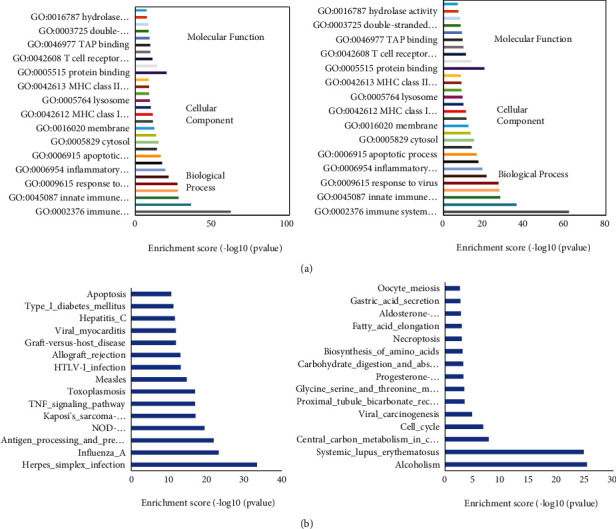
GO terms and KEGG pathway enrichment analysis of upregulated and downregulated mRNAs. (a) Top 10 biological processes, cellular component, and molecular function of GO terms for the analysis of upregulated (left) and downregulated (right) mRNAs. (b) Top 15 KEGG pathways for analysis of upregulated (left) and downregulated (right) mRNAs.

GO functional enrichment analysis revealed that the genes upregulated upon cytokine stimulation are involved with 684 “biological processes,” 99 “cellular components,” and 184 “molecular functions,” while the downregulated genes are involved with 576 “biological processes,” 146 “cellular components,” and 181 “molecular functions”. As shown in Figures 2(a) and 2(b), the top 10 significant “biological processes,” “cellular components,” and “molecular functions” for upregulated mRNAs identified by GO enrichment analysis include “immune system process,” “inflammatory response,” “innate immune response,” “cellular response to IFN-*γ*,” “apoptotic process,” “cytosol,” and “protein binding”; and the top 10 for downregulated mRNAs include “cell cycle,” “mitotic cytokinesis,” “cytoplasm,” “histone binding,” and “protein binding.” We also conducted KEGG analysis to summarize the signaling pathways that these differentially expressed genes were involved with. Data revealed that the upregulated genes were engaged in 86 signaling pathways, whereas the downregulated genes were involved in 50 signaling pathways. The top enrichment results are shown in Figures 2(c) and 2(d). KEGG analysis indicated that upregulated genes in MIN6 cells under proinflammatory cytokine stimulation were primarily involved in the “Herpes simplex virus infection,” “antigen processing and presentation,” “TNF signaling pathway,” and “type 1 diabetes,” whereas downregulated genes were related to “cell cycle,” “necroptosis,” and “Rap1 signaling pathway”.

### 3.3. Validation for the Expression of Significantly Aberrant mRNAs in Pancreatic *β*-Cells Stimulated with Proinflammatory Cytokines by qRT-PCR

**Figure 3:**
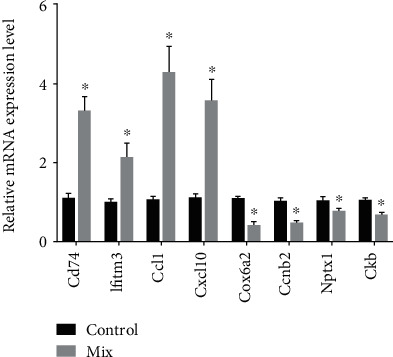
Validation for the expression of eight dysregulated mRNAs. MIN6 cells were incubated in the absence or presence of 2.5 ng/mL IL-1*β*, 2.5 ng/mL TNF-*α*, and 25 ng/mL IFN-*γ* (24 h incubation). The expression of the eight selected mRNAs was measured by qRT-PCR. The results are shown as the means ± SD for three independent experiments. ^∗^*P* < 0.05 was considered statistically significant.

We selected 8 mRNAs to verify the RNA sequencing results by qRT-PCR assays. The results showed an elevated expression of *Cd74*, *Ifitm3*, *Ccl2*, and *Cxcl10* upon the stimulation of proinflammatory cytokines, as well as reduced expression of *Cox6a2*, *Ccnb2*, *Nptx1*, and *Ckb* (Figure 3), which is consistent with the RNA sequencing data. The findings give us the hint that these mRNAs could exert a critical role in proinflammatory cytokine-induced pancreatic *β*-cell destruction.

### 3.4. PPI Network in Pancreatic *β* Cells Stimulated with Proinflammatory Cytokines

**Figure 4:**
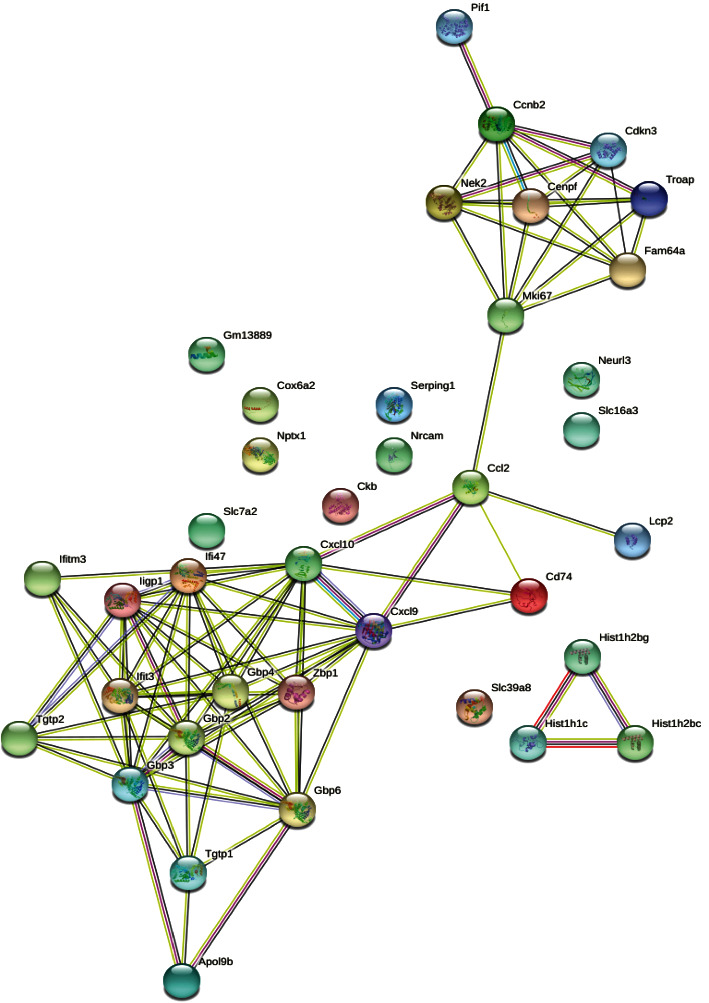
Protein-protein interaction network analysis of top 20 upregulated and 20 downregulated mRNAs was performed by using online tool String (https://www.string-db.org/). The different circles represent different protein-coding genes.

It is important to develop an overall understanding to the interactions among proteins in the process of proinflammatory cytokine-induced pancreatic *β*-cell destruction. Therefore, we selected top 20 upregulated and 20 downregulated mRNAs, respectively, to analyze the interrelation of their coding proteins. The results of the PPI analysis are shown in Figure 4.

### 3.5. circRNA-mRNA Coexpression Networks

**Figure 5:**
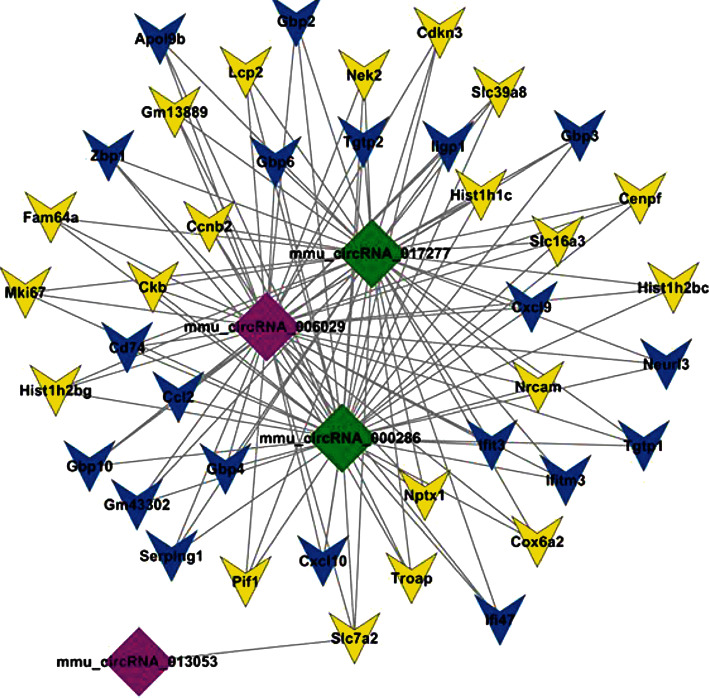
circRNA-mRNA coexpression networks. The four circRNAs (circRNA 006029 and 013053; and circRNA 000286 and 017277) were applied for the coexpression analysis with the top 20 upregulated and downregulated mRNAs. The construction of circRNA-mRNA coexpression network was visualized using Cytoscape. Red diamond represents upregulated circRNA. Green diamond represents downregulated circRNA. Blue VEE represents upregulated mRNA. Yellow VEE represents downregulated mRNA.

circRNAs are a novel class of endogenous ncRNA which regulate gene expression at different levels, from epigenetic gene silencing to posttranscriptional regulation of mRNA stability. They can also function as miRNA sponges, thereby inhibiting miRNA function. Our previous finding reported a group of dysregulated circRNAs in MIN6 cells with the stimulation of proinflammatory cytokines, including upregulated circRNA 006029 and 013053 and downregulated circRNA 000286 and 017277 [23]. Here, the four circRNAs were applied for constructing circRNA-mRNA coexpression network. Based on the network, we identified impossible regulatory relationship between circRNAs and mRNAs (Figure 5). From the data, we observed that upregulated circRNA 006029 and downregulated circRNA 000286 and 017277 were associated with the vast majority of selected dysregulated mRNAs, while circRNA 013053 was only related to the protein-coding gene, *Slc7a2*. These data gave us the hint that circRNAs might regulate mRNAs, thereby contributing to the development of T1DM.

## 4. Discussion

T1DM is an autoimmune disease characterized by pancreatic *β*-cell dysfunction mediated by autoimmune responses [24, 25]. Insulitis, an inflammatory process where the pancreatic islets are infiltrated by immune cells, is a dominant pathological event during T1DM progression [1]. During insulitis, prolonged exposure of *β*-cells to proinflammatory cytokines such as IL-1*β*, TNF-*α*, and IFN-*γ* will modulate the expression of many genes, thereby resulting in severe impairment of key signaling pathways and *β*-cell functions [1–3]. Herein, we attempted to identify the changes of mRNA expression profiles in MIN6 cells exposed to proinflammatory cytokines IL-1*β*, TNF-*α*, and IFN-*γ*, and to analyze the aberrant mRNA expression patterns in the process.

Over the years, RNA sequencing technology has been emerging as a novel and promising tool for transcriptomic studies, which exhibits high reproducibility and low frequency of false positives [26, 27]. Compared to cDNA microarrays, RNA sequencing technology is capable of identifying more genes, Moreover, it allows identifications of both whole genes and splice variants. By using RNA sequencing, we identified 809 upregulated and 946 downregulated mRNAs in cytokine-stimulated cells compared with the control group. This was furtherly proved by qRT-PCR showing the dysregulations of multiple mRNAs. These data showed aberrant mRNA expression patterns in the MIN6 cells stimulated with the proinflammatory cytokines IL-1*β*, TNF-*α*, and IFN-*γ*.

Interestingly, as we analyzed these dysregulated mRNAs in cytokine-treated MIN6 cells, we found that some of these dysregulated mRNAs are participated in inflammatory responses (i.e., *Ccl2*, *Ccl5*, *Ccl8*, *Ccl20*, *Cxcl10*, *Cxcl2*, and *Icam1*), IFN-*γ* signaling (i.e., *Irf1*, *Irf2*, *Irf5*, *Irf7*, *Igtp*, *Stat1*, *Stat2*, *Stat3*, and *Jak2*), NF-*κ*B regulation (i.e., *Nfkb1*, *Nfkbia*, *Nfkbib*, *Nfkb2*, and *Nfkbiz*), and ER stress and apoptosis (i.e., *Atf3*, *Bid*, *Casp1*, *Casp4*, and *Ddit3*). Through systematic analysis, the top 10 of each GO term include “immune system process,” “inflammatory response,” “innate immune response,” “cellular response to IFN-*γ*,” “apoptotic process,” “cytosol,” and “protein binding”; and the top 15 of KEGG pathways include “antigen processing and presentation,” “TNF signaling pathway” and “type 1 diabetes”. These data were in concordance with previous findings in INS1E cells [28], rat *β*-cells [4], mouse islets [29], and human islets [30], suggesting an essential engagement of these dysregulated mRNA molecules in the process of proinflammatory cytokine-induced *β*-cell destruction. We also observed the association of protein-coding mRNAs by the construction of PPI networks. It will give us the hint of the interaction of proteins in the development of type 1 diabetes and help us to understand the pathogenesis of T1D.

The changed expression of these protein-coding genes in *β*-cells stimulated with proinflammatory cytokines is modulated through many ways. As afore clarified, activated signaling pathways are an important mechanism regulating the downstream gene expression and causing *β*-cell damage, such as NF-*κ*B, STAT3, and ER stress [6]. Apart from this, ncRNAs, including miRNA, lncRNA, and circRNA, have also been demonstrated to regulate gene expression in this process [7, 9, 11]. Previous studies have shown a changed miRNA expression pattern in proinflammatory cytokine-induced *β*-cell destruction [12]. Thus, one can speculate that changed miRNAs might participate in the regulations of mRNA expression, subsequently causing *β*-cell dysfunction. Motterle et al. and Sun et al. reported key or partial roles that the differentially expressed lncRNA may serve in pancreatic *β*-cell dysfunctions in response to proinflammatory cytokines [5, 19]. These lncRNAs may play key or partial roles in cytokine-mediated *β*-cell dysfunction by regulating mRNA expression. Moreover, by using the circRNA microarray data in our previous study [23], we constructed a circRNA-mRNA coexpression network and obtained that circRNAs may be implicated in *β*-cell dysfunction. Therefore, we speculate that the regulation of ncRNAs contributes to the changed mRNAs in proinflammatory cytokine-induced *β*-cell destruction. In any event, specific roles of these ncRNAs in *β*-cells require to be further elucidated, and expression levels of ncRNAs in individuals with T1DM need to be investigated in depth in the future as well.

## 5. Conclusion

Our current study reveals many differentially expressed mRNAs in cytokine-induced MIN6 cell destruction by using RNA sequencing. These differentially expressed mRNAs may play key or partial roles in cytokine-mediated *β*-cell dysfunction. And we also analyze the regulation of mRNAs by circRNAs. Understanding the functions and regulatory mechanisms of these mRNAs could help us to identify new diagnostic and therapy targets for T1DM. Thus, further studies to identify the contributions of some interested targets in the pathogenesis of T1DM based on current study would be important and valuable.

## Figures and Tables

**Table 1:** The top 20 upregulated and downregulated mRNAs.

Upregulated mRNA	log2 (fold change)	Downregulated mRNA	log2 (fold change)
*Gbp2*	8.454	*Cox6a2*	-2.422
*Cd74*	8.091	*Gm13889*	-2.395
*Ifitm3*	8.008	*Slc16a3*	-2.208
*Ifi47*	7.497	*Hist1h1c*	-2.106
*Gbp6*	7.391	*Lcp2*	-2.091
*Iigp1*	7.332	*Ccnb2*	-2.062
*Serping1*	7.246	*Mki67*	-2.055
*Ifit3*	7.166	*Nptx1*	-2.039
*Gbp10*	7.142	*Cdkn3*	-1.983
*Cxcl10*	7.11	*Slc39a8*	-1.923
*Zbp1*	7.019	*Hist1h2bc*	-1.901
*Gbp3*	7.015	*Pif1*	-1.847
*Cxcl9*	6.948	*Nrcam*	-1.824
*Gbp4*	6.94	*Cenpf*	-1.823
*Tgtp2*	6.905	*Hist1h2bg*	-1.818
*Ccl2*	6.854	*Ckb*	-1.817
*Tgtp1*	6.822	*Troap*	-1.804
*Gm43302*	6.654	*Nek2*	-1.799
*Neurl3*	6.615	*Fam64a*	-1.795
*Apol9b*	6.586	*Slc7a2*	-1.791

## Data Availability

The data relative to this manuscript can be available by emailing to Dr. Ying Zheng.
